# Pd:In-Doped TiO_2_ as a Bifunctional Catalyst for the Photoelectrochemical Oxidation of Paracetamol and Simultaneous Green Hydrogen Production

**DOI:** 10.3390/molecules29051073

**Published:** 2024-02-29

**Authors:** Nicolás Sacco, Alexander Iguini, Ilaria Gamba, Fernanda Albana Marchesini, Gonzalo García

**Affiliations:** 1 Instituto de Investigaciones en Catálisis y Petroquímica, INCAPE (UNL-CONICET), Facultad de Ingeniería Química, Santiago del Estero 2829, Santa Fe 3000, Argentina; nsacco@fiq.unl.edu.ar (N.S.); albana.marchesini@gmail.com (F.A.M.); 2 Departamento de Química, Instituto Universitario de Materiales y Nanotecnología, Universidad de La Laguna (ULL), P.O. Box 456, 38200 La Laguna, Spain; alu0101100890@ull.edu.es (A.I.); ilgamba@ull.edu.es (I.G.)

**Keywords:** PdIn-doped TiO_2_ catalyst, green H_2_ production, photoelectrochemical oxidation, paracetamol, pharmaceutical removal from water

## Abstract

The integration of clean energy generation with wastewater treatment holds promise for addressing both environmental and energy concerns. Focusing on photocatalytic hydrogen production and wastewater treatment, this study introduces PdIn/TiO_2_ catalysts for the simultaneous removal of the pharmaceutical contaminant paracetamol (PTM) and hydrogen production. Physicochemical characterization showed a high distribution of Pd and In on the support as well as a high interaction with it. The Pd and In deposition enhance the light absorption capability and significantly improve the hydrogen evolution reaction (HER) in the absence and presence of paracetamol compared to TiO_2_. On the other hand, the photoelectroxidation of PTM at TiO_2_ and PdIn/TiO_2_ follows the full mineralization path and, accordingly, is limited by the adsorption of intermediate species on the electrode surface. Thus, PdIn-doped TiO_2_ stands out as a promising photoelectrocatalyst, showcasing enhanced physicochemical properties and superior photoelectrocatalytic performance. This underscores its potential for both environmental remediation and sustainable hydrogen production.

## 1. Introduction

Energy production and water availability pose significant challenges for future generations. The global consumption of both resources is experiencing substantial growth, driven by population increases and improved living standards. The combustion of fossil fuels releases greenhouse gases and other pollutants into the atmosphere, resulting in critical consequences for the environment. On the other hand, water availability is threatened by the presence of contaminants in wastewater and the lack of water sanitation solutions.

The Sustainable Development Goals (SDGs) set forth by the United Nations for 2030 include specific objectives related to energy consumption (SDG 7: Affordable and Clean Energy) and water pollution (SDG 6: Clean Water Sanitation). Renewable energies could be a key part of the solution for sustainable energy production if electricity storage is ensured. Hydrogen has emerged as a highly promising renewable fuel due to its high energy content, lack of environmental hazards, and, most importantly, its ability to be produced from water [1,2]. The production of green hydrogen is a promising way to supply and distribute intermittently generated energy through fuel cells. However, its production through water splitting is not cost-effective due to the large amounts of energy required for the oxygen evolution reaction (OER) at the anode. It is important to consider that in the case of water splitting, the thermodynamic potential needed to break down water into oxygen and hydrogen is 1.23 V. However, high overpotentials are usually employed to overcome the slow kinetics of the OER [3]. Photoelectrocatalytic production of hydrogen by oxidizing organic or inorganic compounds at the anode can be achieved without the need for electrical input, but current catalysts do not meet the requirements to approach viability goals. On the other hand, the potential required for the degradation of contaminants depends on the nature and concentration of the contaminants, the nature of the photoelectrocatalyst, and the efficiency of the process used [4].

Electrocatalytic hydrogen production has emerged as a popular method for hydrogen generation [5]. However, there are several issues, including cost-effectiveness, associated with these techniques. To address the dual challenges of the energy crisis and environmental pollution, sustainable photocatalytic hydrogen production has shown promise [6,7]. However, efficient photocatalytic hydrogen generation typically requires the use of external sacrificial agents or donors, such as alcohols or organic acids, to scavenge holes and reduce recombination [8]. The addition of these sacrificial agents increases the cost of hydrogen evolution, making it economically viable but less practical in the long run [9]. Thus, for sustainable and efficient hydrogen production, there are two main requirements to achieve. Firstly, the photocatalyst must possess efficient electron–hole separation, numerous active reaction sites, and high visible light activity [10,11], which is crucial for effective hydrogen generation. The second challenge involves the recovery or generation of hydrogen energy from wastewater, enabling environmentally friendly and sustainable energy production combined with water treatment on a larger scale. This integrated approach holds promise for addressing both energy and environmental concerns. 

Various catalysts for efficient photocatalytic H_2_ generation have been synthesized by different authors. Meng et al. [12] developed Ni_12_P_5_/ZnIn_2_S_4_ (NP/ZIS) heterostructures using a hydrothermal method, demonstrating visible-light-driven photocatalytic splitting of benzyl alcohol into H_2_ and benzaldehyde. The use of 7% NP/ZIS significantly improved the thermodynamics and kinetics of H_2_ production compared to pure water splitting and individual ZIS, attributed to increased surface area, porous structure, creation of defect states (zinc vacancies), and the enhancement of the NP co-catalyst. Amorphous TiO_2_ and Co-ZnIn_2_S_4_ were combined to form a heterojunction, improving photocarrier separation efficiency and catalyst stability. The introduction of amorphous TiO_2_ induced oxygen vacancies, enhancing carrier density. Additionally, MoP nanoparticles were introduced as co-catalysts, serving as hydrogen production sites and achieving efficient hydrogen production [13]. 

Long et al. [14] investigated nanostructured polymeric carbon nitride (PCN) for visible-light-driven photocatalytic hydrogen evolution, attributing improved activity to increased BET specific surface area, higher active site quantity, and accelerated transfer and separation of photo-excited charge carriers. Additionally, Zheng et al. [15] demonstrated the excellent photocatalytic activity of Au/ZnO nanomaterial in bisphenol A degradation and photoelectrochemical water splitting. The enhanced activities were linked to heightened light absorption and unique charge transfer of photogenerated electrons, effectively reducing the recombination rate and prolonging the lifetime of photo-excited carriers.

Contaminants of emerging concern (CECs) are increasingly being detected in water sources worldwide, posing significant challenges to water quality and human health. These CECs include a wide range of pollutants, such as pharmaceuticals, personal care products, pesticides, industrial chemicals, and microplastics, which can enter water bodies through various pathways [16,17]. The presence of CECs in water raises concerns for both ecological and human health. These contaminants can have adverse effects on aquatic ecosystems, including the disruption of endocrine systems, alteration of reproductive behaviors, and changes in the composition of microbial communities. In terms of human health, exposure to CECs through drinking water consumption or recreational activities in contaminated water bodies can pose risks, particularly for vulnerable populations such as children and pregnant women [18].

Paracetamol, also known as acetaminophen, is a widely used over-the-counter medication for pain relief and fever reduction. Like many pharmaceuticals, it can enter the environment through various pathways, including improper disposal, excretion, and wastewater treatment plant effluents. While it is generally considered safe for human use when taken at recommended doses, the presence of PTM in water bodies as a CEC is a topic of growing interest and research. The presence of acetaminophen in aquatic environments can have adverse effects on aquatic organisms. Even at low concentrations, it can disrupt the endocrine systems of fish and other aquatic organisms, affecting their reproductive capabilities [19].

Thus, utilizing PTM as a sacrificial agent in photocatalytic hydrogen evolution serves the purposes of both clean energy generation and wastewater treatment.

Photoelectrochemical oxidation (PECO) is indeed a promising technique for the removal of PTM from water. PECO involves the use of a photoactive electrode, typically a semiconductor material, which generates reactive oxygen species (ROS) upon exposure to light. These ROS, such as hydroxyl radicals, play a crucial role in the degradation of organic contaminants like PTM [20].

Since the discovery of TiO_2_’s ability for water-splitting and photocatalytic degradation of organic compounds, numerous semiconductors have been studied for environmental and energy applications. TiO_2_ is the most extensively investigated due to its chemical stability, low cost, and good photocatalytic efficiency [21]. However, TiO_2_ does have a limitation in its optical response. With a large band gap (E.g., ~3.2 eV), TiO_2_ primarily responds to UV light, which accounts for only 5% of solar energy [22]. To address this issue, various modifications of TiO_2_ have been extensively studied to enhance its wavelength range response, promote charge generation, and facilitate efficient charge separation to minimize recombination [23]. Techniques for TiO_2_ modifications include metal loading, ion doping, semiconductor coupling, and dye sensitization. Depositing precious metals or rare-earth metals onto semiconductors is a widely investigated approach to enhance the photocatalytic properties of TiO_2_ [24]. This method offers two main advantages: the formation of a Schottky junction for efficient charge separation and the localized surface plasmon resonance (LSPR) effect, which promotes enhanced charge generation through the absorption of visible light.

Pd and Pd-In catalysts have been widely reported in the catalytic reduction and electrochemical reduction of inorganic ions present in water [25]. It has been demonstrated that the Pd-In combination can hydrogenate nitrate ions into nitrites in water [26]. On the other hand, Pd has attracted significant attention as it is one of the platinum-group metals with high catalytic activity for the HER [27]. It is important to note that most of the catalysts, either mono- or bimetallic, based on Pd for the electrocatalytic production of H_2_ imply the use of high metal loadings, which considerably increases the cost of these technologies. This work evaluates the catalytic performance of PdIn-doped TiO_2_ catalysts (Pd, 1 wt.%, In 0.25 wt.%) in the photoelectrochemical oxidation of PTM and the simultaneous HER. The reaction mechanism, both for PTM oxidation and HER, and the stability of the catalyst are discussed.

## 2. Results and Discussion

### 2.1. Characterization

#### 2.1.1. UV-DRS Analysis

**Figure 1:**
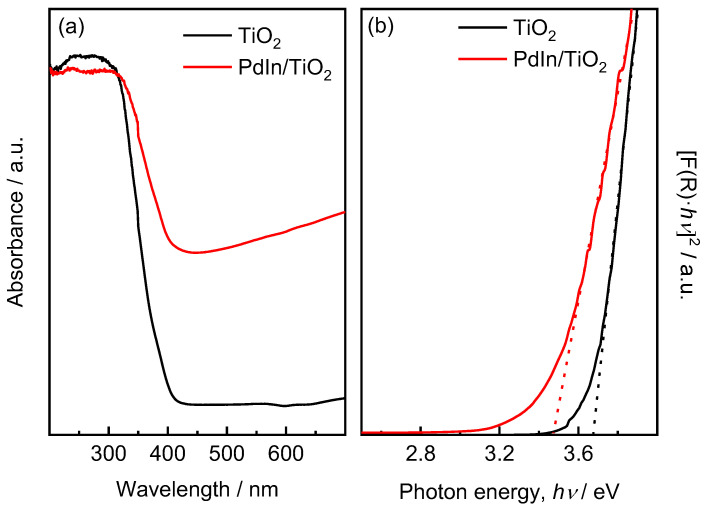
(**a**) UV–vis absorption spectra and (**b**) bandgap energy plot (Kubelka–Munk function) of TiO_2_ (black line) and PdIn/TiO_2_ (red line) materials.

To evaluate the absorbance properties of TiO_2_ and PdIn/TiO_2_ synthesized in this study, UV–Vis diffuse reflectance spectra (DRS) were measured (Figure 1). The absorption band edge of TiO_2_ occurs at approximately 400 nm. The addition of PdIn leads to an increase in absorption at longer wavelengths within the visible range. The band gap values of TiO_2_ and PdIn/TiO_2_ were 3.67 and 3.47 eV, respectively. The lowest band gap value obtained after the impregnation of PdIn onto TiO_2_ would indicate that the Pd and In deposition enhances the light absorption capability, resulting in a possible higher catalytic activity when compared to TiO_2_ alone. This behavior could be related to the Fermi levels of Pd, which are lower than those of TiO_2_, facilitating the efficient transfer of photogenerated electrons from the conduction band of TiO_2_ to the metal particles. This process of electron trapping greatly diminishes the rate of electron–hole recombination, leading to enhanced photocatalytic reactions.

#### 2.1.2. Physicochemical Properties

**Figure 2:**
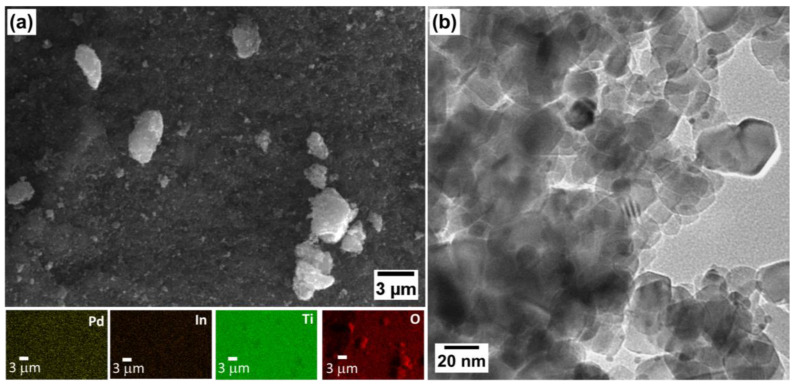
(**a**) SEM micrograph of PdIn/TiO_2_ catalyst and corresponding mappings of Pd, In, O, and Ti species. (**b**) HRTEM micrograph of PdIn/TiO_2_ catalyst.

Morphology and elemental analysis of PdIn/TiO_2_ were studied through SEM–EDS and HRTEM techniques, respectively. Figure 2a shows an SEM–EDS micrograph of the PdIn/TiO_2_ catalyst and the corresponding mappings. A homogeneous distribution of the materials was obtained. Indeed, a high distribution of Pd and In on the TiO_2_ particles is perceived, with an average wt.% composition of 0.85 ± 0.07 and 0.22 ± 0.04, respectively, and a Pd:In atomic ratio close to the nominal value was detected. Figure 2b shows the HRTEM micrograph of the PdIn/TiO_2_ catalyst. Particles with an average size of 18 nm are observed and depicted in the particle size distribution graph (Figure S1). Furthermore, particles with smaller sizes (~5 nm) are discerned and may be ascribed to Pd and/or In-based species.

The surface area and pore volume of PdIn/TiO_2_, obtained from BET analysis, were 48.84 m^2^/g and 0.0015 cm^3^/g, respectively. For commercial TiO_2_, a surface area of 48.47 m^2^/g was reported, a similar value [28]. Therefore, no diminution of the surface area is perceived after metal deposition onto TiO_2_ material. Figure S2 shows nitrogen adsorption isotherms of TiO_2_ and PdIn/TiO_2_ materials. N_2_ adsorption isotherms align with type II, as per the IUPAC classification. These isotherms are indicative of non-porous or macroporous solids, exhibiting low or negligible microporosity and unrestricted multilayer adsorption.

**Figure 3:**
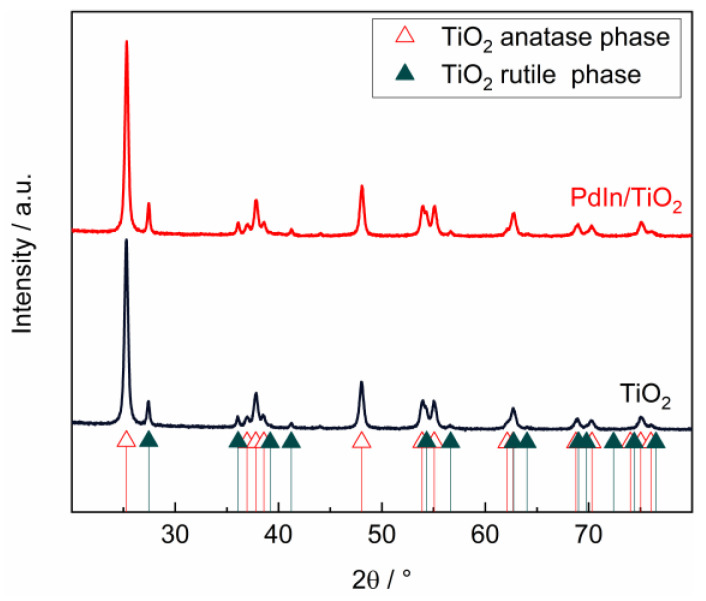
XRD patterns of PdIn/TiO_2_ (red line) and TiO_2_ (black line) samples.

XRD patterns of PdIn/TiO_2_ and the bare support were assayed to elucidate the crystalline phases present. Figure 3 shows the corresponding diffractograms, respectively. Anatase (JCPDS 00-021-1272) and rutile (JCPDS 00-021-1276) phases were detected in both TiO_2_ and PdIn/TiO_2_ catalysts. No crystalline phases corresponding to Pd or In were detected, which could be due to the low metal loading, the high dispersion of the material on the support, and/or the amorphous nature of the dispersed species. The crystallite size of both phases, anatase (plane 1 0 1, 2θ = 25.281°) and rutile (plane 1 1 0, 2θ = 27.477°), for PdIn/TiO_2_ and TiO_2_ were calculated using the Scherrer equation. Crystallite sizes of 20.93 nm were obtained for the anatase phase of both catalysts. For the rutile phase of TiO_2_ and PdIn/TiO_2_, crystallite sizes of 25.6 and 31.4 nm, respectively, were obtained. This change of crystallite size in the rutile phase could be due to a strong interaction with Pd and In species or to their introduction into its crystalline network since the doping of the material could affect its electronic and structural properties and consequently the crystallite size [29,30,31].

The electroactive surface area was calculated by performing CVs at different scan rates in a potential range where no faradaic reaction occurs (i.e., capacitive currents are employed) by plotting the anodic and cathodic current densities at a fixed potential versus the scanning rate (see Figure S3 as an example for GC in the Supplementary Material). The same procedure was performed for TiO_2_ and PdIn/TiO_2_. The corresponding values of anodic and cathodic electrochemical double-layer capacitances (EDLC_A_ and EDLC_V_, respectively) and electroactive surface areas for different electrodes, calculated using Equations (14) and (15) (see Experimental section), are summarized in Table 1. Considering the slopes obtained for each material, it can be observed that the PdIn/TiO_2_ catalyst reveals a higher ECSA than GC and TiO_2_.

### 2.2. Hydrogen Evolution Reaction

**Figure 4:**
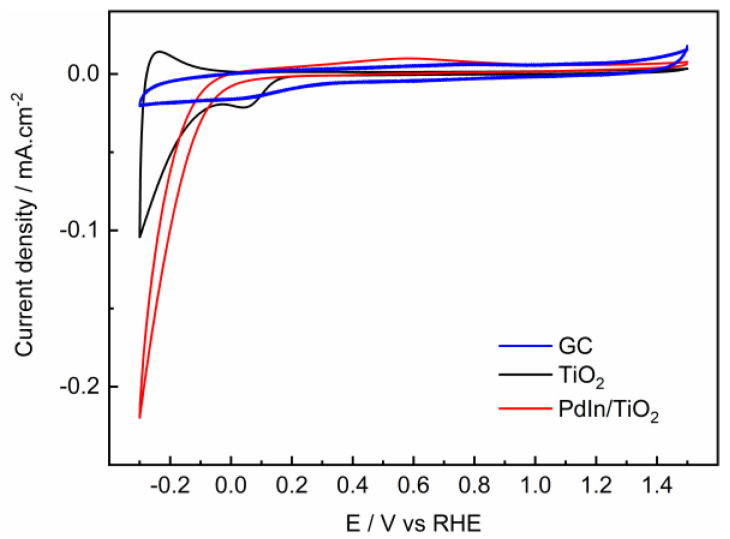
Cyclic voltammograms of GC (blue line), TiO_2_ (black line), and PdIn/TiO_2_ (red line). Sweep rate = 20 mV·s^−1^, in 0.1 M phosphate buffer solution, pH = 7.

Figure 4 shows cyclic voltammograms performed at GC (black line), TiO_2_ (red line), and PdIn/TiO_2_ (blue line) between −0.3 V and 1.5 V in the electrolyte solution. As expected, the GC electrode reveals only capacitive currents in the potential range under study. On the other hand, TiO_2_ and PdIn/TiO_2_ catalysts show an increment of the cathodic current at potentials more negative than 0.0 V, which is associated with the HER. Interestingly, the presence of PdIn significantly improves the HER in the electrolyte solution. Indeed, PdIn/TiO_2_ develops double the current at −0.25 V compared with TiO_2_.

**Figure 5:**
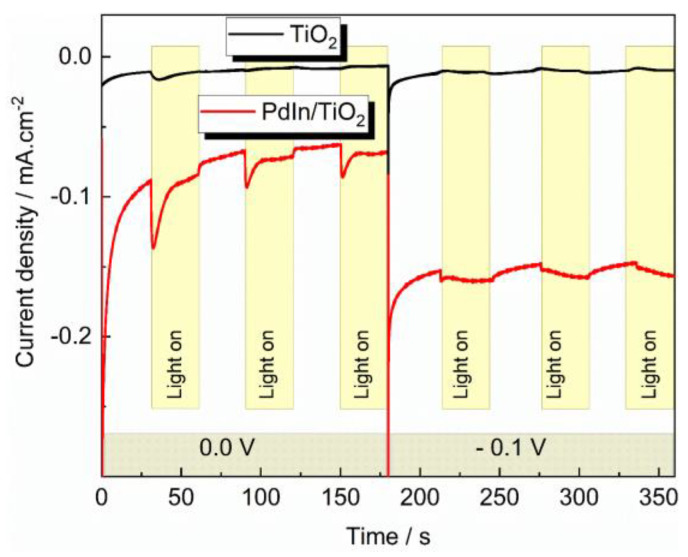
Current transients of TiO_2_ (black line) and PdIn/TiO_2_ (red line) recorded at 0.0 and −0.1 V in 0.1 M phosphate buffer solution, pH = 7, under the absence and presence of light.

The photoelectrocatalytic performance of TiO_2_ (black lines) and PdIn/TiO_2_ (red lines) catalysts toward the HER was evaluated through chronoamperometry technique in the presence (light on) and absence (light of) of radiation at 0 and −0.1 V with an irradiation intermittence of 30 s (Figure 5).

TiO_2_ material develops low cathodic current and photocurrents at both studied potentials. Oppositely, the PdIn/TiO_2_ catalyst reveals high cathodic current values at both applied potentials and suitable photoactivity at 0.0 V. The decrease of the photoactivity of PdIn/TiO_2_ at −0.1 V suggests that the flat band potential is close. In addition, the PdIn/TiO_2_ catalyst develops similar cathodic current values over time, which indicates an appropriate photoelectrochemical stability and, thus, a suitable photoelectrocatalytic performance toward the HER.

A Tafel plot was employed to better understand the reaction kinetics and mechanism of the HER at the best catalyst developed in the current work. For this purpose, linear sweep voltammetry (LSV) was performed between 0.2 and −0.2 V at a sweep speed of 5 mV·s^−1^. Two reaction mechanisms are commonly discussed in the literature [32,33], denoted as Volmer–Heyrovsky and Volmer–Tafel. Both mechanisms have in common that hydrogen is adsorbed (H_ad_) on the electrode through the electrochemical Volmer step but differ in the second stage. For the Volmer–Heyrovsky mechanism (Equation (1)), the Heyrovsky step (Equation (2)) involves the adsorbed hydrogen recombining with another proton from the solution to release an H_2_ molecule. On the other hand, the Volmer–Tafel mechanism consists of two consecutive Volmer steps and the Tafel step (Equation (3)) in a recombination step of two adjacent hydrogen adsorbates to form H_2_.

(1) $$Volmer:H_{2}O+e^{-}⇌H_{ad}+{OH}^{-},$$

(2) $$Heyrovsky:H_{ad}+H_{2}O+e^{-}⇌H_{2}+{OH}^{-},$$

and

(3) $$Tafel:H_{ad}+H_{ad}⇌H_{2}$$

**Figure 6:**
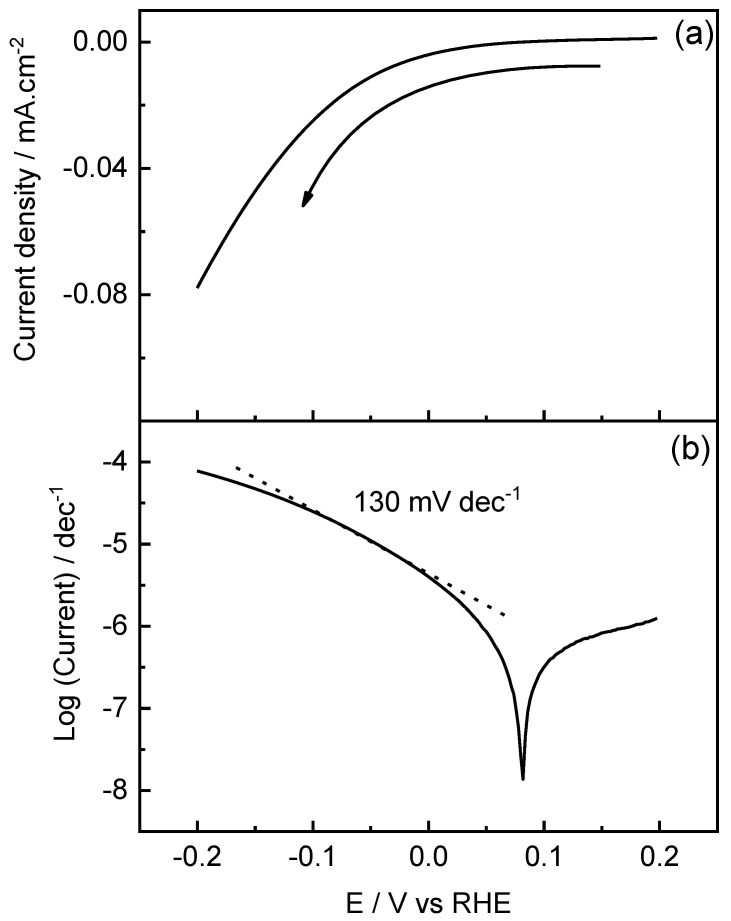
Linear sweep voltammogram recorded at 5  mV·s^−1^ (**a**) and Tafel plot (**b**) for PdIn/TiO_2_ in 0.1 M phosphate buffer solution, pH = 7.

Tafel slope (TS) values were employed to discern which reaction mechanism follows the HER at the PdIn/TiO_2_ catalyst. TS values of 120, 30, and 40 mV·dec^−1^ are associated with Volmer, Tafel, and Heyrovsky as the rate-determining step (RDS), respectively. Figure 6a shows the LSV recorded for PdIn/TiO_2_ performed at 5 mv·s^−1^ from 0.2 V to −0.2 V in the electrolyte solution. Figure 6b shows a TS close to 120 mV·dec^−1^, which is attributed to the Volmer step as the RDS during the HER at the PdIn/TiO_2_ catalyst. In this sense, the high TS may be attributed to the high amount of surface oxygenated species of TiO_2_, which may inhibit the first electron transfer step. 

### 2.3. Paracetamol Oxidation Reaction

**Figure 7:**
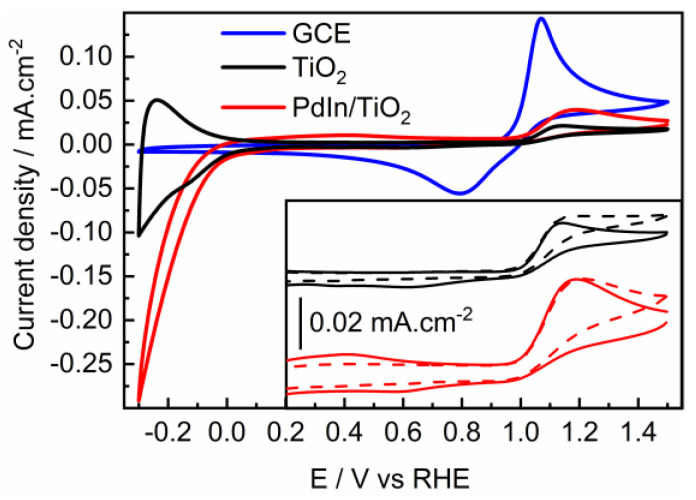
Cyclic voltammograms of GC (blue line), TiO_2_ (black line), and PdIn/TiO_2_ (red line) in a 100 ppm PTM solution in 0.1 M phosphate buffer solution. Sweep rate = 20 mV·s^−1^, pH = 7. Inset (For the sake of clarity, the CVs were vertically translated): TiO_2_ and PdIn/TiO_2_ in the absence (solid lines) and the presence of light (dashed lines) in a 100 ppm PTM solution in 0.1 M phosphate buffer solution. Sweep rate = 20 mV·s^−1^, pH = 7.

The photoelectrocatalytic activity of GCE, PdIn/TiO_2_, and TiO_2_ support towards the oxidation of PTM (100 ppm) was evaluated using cyclic voltammetry under irradiation and in the absence of irradiation. Figure 7 shows CV profiles of PTM electro-oxidation in the dark at GCE, TiO_2_, and PdIn/TiO_2_. As discussed above (see Figure 4), the presence of PTM does not change the catalytic performance toward the HER at PdIn/TiO_2_, and consequently, the catalytic active sites for the HER are not compromised.

PTM oxidation on GCE (blue line) exhibits an anodic current generation with an anodic peak at 1.1V, and an onset potential of 1.0 V. A quasi-reversible process with a peak-to-peak separation of ΔV = 250 mV was determined, as reported by Nematollahi et al. [34] for the same material and similar pH conditions. At more positive potentials than the anodic current peak, a large drop in current density is observed, showing a Cottrell behavior, indicating that the process is limited by diffusion of the species towards the electrode surface.

On the other hand, TiO_2_ (black line) and PdIn/TiO_2_ (red line) show an irreversible behavior toward the PTM oxidation with onset potentials of 1.0 V and ΔV = 750 and 420 mV, respectively. Evidently, at higher potentials than the anodic peak current, the oxidation behavior is different for GCE compared with TiO_2_-based materials. This suggests that the reaction mechanism at TiO_2_-based materials is limited by adsorbed species.

The same CV experiments were performed on TiO_2_ and PdIn/TiO_2_ but in the presence of light. The inset plot in Figure 7 compares voltammograms corresponding to the PTM oxidation at TiO_2_ and PdIn/TiO_2_ catalysts under the absence (solid lines) and the presence (dashed lines) of light. During the oxidation of PTM in the absence of light, at more positive potentials than the anodic peak, the current density slightly decreases (i.e., non-Cottrell behavior) with the rise of the applied potential, suggesting that the current is limited by kinetic. Conversely, in the presence of light, the current density remained almost constant at more positive potentials than the anodic peak, which implies that the current is limited by kinetics and suggests that adsorbed species are responsible. On the other hand, during the reverse scan, the presence of light made the system completely irreversible, i.e., no cathodic currents were discerned. 

To better understand the kinetics and reaction mechanism of the PTM oxidation at all materials studied in the current work, rotating disk experiments at different rotational speeds were performed.

**Figure 8:**
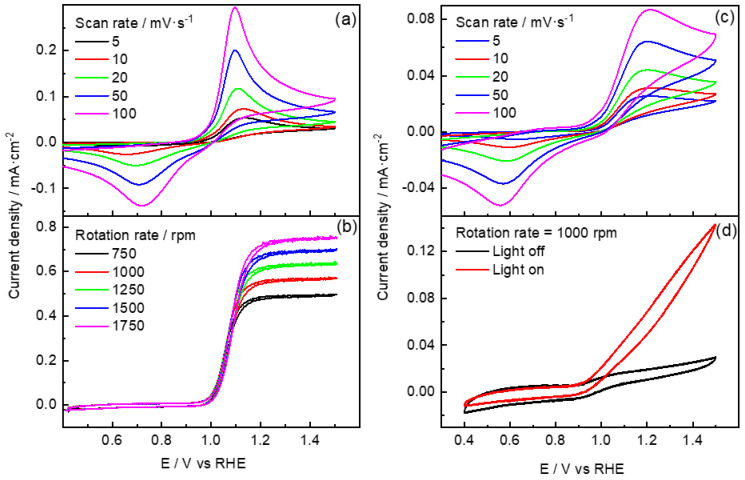
Cyclic voltammograms at diverse sweep rates at (**a**) GCE and (**c**) PdIn/TiO_2_. Steady-state polarization curves recorded at 10 mV·s^−1^ at several rotation rates at (**b**) GCE and (**d**) at the PdIn/TiO_2_ electrode in the presence (red line) and the absence (black line) of radiation. All assays were performed in a 100 ppm PTM solution in 0.1 M phosphate buffer, pH = 7.

Figure 8a,b compares CV profiles of PTM oxidation at GCE performed at different sweep rates and rotational rates, respectively. These experiments demonstrate that the PTM oxidation process is diffusion-limited on the GCE since, as shown in Figure 8, the anodic current density reaches a constant diffusion value (I_DIF_), which increases with the growth of the rotational speed.

For GCE, Randles–Sevsick and Koutecky–Levich plots with the corresponding slope value are shown in Figure S4, respectively. Koutecky–Levich equation is shown in Equation (4), where $I_{DIF}$ is the limit current (A), $I_{k}\;$ the kinetic current, and $I_{lev}$ is expressed using Equation (5):

(4) $$\frac{1}{I_{DIF}}=\frac{1}{I_{lev}}+\frac{1}{I_{k}}\;$$

and

(5) $$I_{lev}=0.62nFAD^{2/3}\omega^{1/2}\nu^{-1/6}C,$$

where *v* is the kinematic viscosity, *w* is the angular frequency of rotation (rad·s^−1^), A is the disk electrode area (cm^2^), and other symbols have their conventional meanings. By plotting $\frac{1}{I_{DIF}}\;vs\;\omega^{-1/2}$ and obtaining from the literature for the kinematic viscosity of the electrolyte (0.012 cm^2^·s^−1^) [35] and the diffusion coefficient *D* (6.1 × 10^−6^ cm^2^·s^−1^) [36], the number of electrons transferred involved in the reaction yielded a value of 2, as reported by Nematollahi et al. [34]. Thus, this process could be associated with the reversible transformation of PTM into N-acetyl-p-benzoquinone amine (NAPQI) [34]:

(6) $$C_{8}H_{9}{\mathrm{NO}}_{2}⇌C_{8}H_{7}{\mathrm{NO}}_{2}+2H^{+}+2e^{-}.$$

Figure 8c,d compares CV profiles of PTM oxidation at PdIn/TiO_2_ performed at different sweep rates and rotational rates, respectively. On the other hand, the anodic peak potential for TiO_2_ and PdIn/TiO_2_ was plotted as a function of the scan rate, and a linear trend was discerned, which suggests that the process is limited by the adsorption of species on the electrode surface. The number of electrons (*n*) transferred to the surface of the electrode was calculated through the Laviron equation for an irreversible process, where α is the electron-transfer coefficient (0.5), and *n* is the number of electrons involved in the redox process [37]:

(7) $$E_{pA}=\frac{RT}{\left(1-\alpha \right)nF}\log\left(v\right).$$

For both TiO_2_-based electrodes, the number of transferred electrons was 1, and the subsequent reaction is the most plausible to occur:

(8) $$C_{8}H_{9}{\mathrm{NO}}_{2}\;⇌(C_{8}H_{8}{\mathrm{NO}}_{2}{)}_{\mathrm{ad}}+H^{+}+1e^{-}.$$

Then, the adsorbed species may follow subsequent reactions at more positive potentials:

(9) $$(C_{8}H_{8}{\mathrm{NO}}_{2}{)}_{\mathrm{ad}}⇌(C_{8}H_{7}{\mathrm{NO}}_{2}{)}_{\mathrm{ad}}+H^{+}+1e^{-},$$

(10) $$(C_{8}H_{7}{\mathrm{NO}}_{2}{)}_{\mathrm{ad}}⇌C_{8}H_{7}{\mathrm{NO}}_{2},$$

and

(11) $$(C_{8}H_{7}{\mathrm{NO}}_{2}{)}_{\mathrm{ad}}+14H_{2}O\;⇀\;8{\mathrm{CO}}_{2}+\frac{1}{2}N_{2}+35H^{+}+35e^{-}.$$

Equation (10) seems to be facile at GCE, while the opposite happens at TiO_2_-based electrodes, and accordingly, the adsorbate path is favored. In this sense, Equation (11) indicates the global reaction toward the total mineralization of paracetamol, which is expected to follow the adsorbate route via deprotonation processes. In this context, it is important to note that the presence of radiation at TiO_2_-based electrodes completely inhibits the pathway toward soluble species (i.e., Equation (10)), and consequently, no cathodic peaks are detected during the reverse sweep. 

In this regard, Figure 8d suggests the aforementioned phenomenon, as a subsequent increment in the anodic current is perceived with the rise of applied potential in the presence of light. Remarkably, the same current values were obtained at rotation rates higher than 750 rpm, and no inhibition was discerned in the subsequent cycles. Therefore, the adsorbate route seems to predominate in TiO_2_-based catalysts. Furthermore, the addition of a small amount of PdIn into TiO_2_ not only increases the catalytic efficiency toward PTM oxidation but also intensely raises the HER, which is not inhibited in the presence of the organic molecule. 

**Figure 9:**
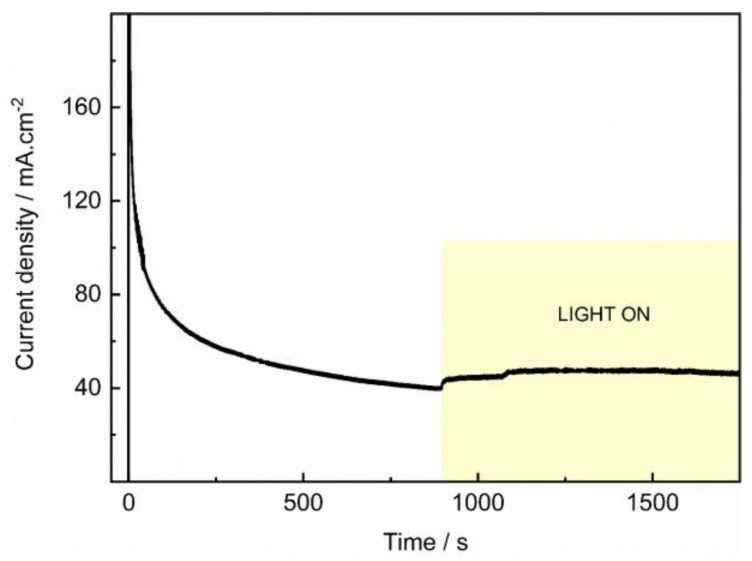
Photo/current transients of PdIn/TiO_2_ recorded at 1.2 V and 1000 rpm, under the absence and the presence of light in a 100 ppm PTM solution in 0.1 M phosphate buffer solution, pH = 7.

Finally, to test the catalytic stability of PdIn/TiO_2_ toward the degradation of PTM in the absence and presence of light, a current transient was recorded at 1.2 V and depicted in Figure 9. An initial decrease in the anodic current density in the absence of light is observed, which rises and remains almost constant when the system is exposed to light. This indicates an improved catalyst performance toward PTM photoelectroxidation.

## 3. Experimental

### 3.1. Catalyst Synthesis

The bimetallic catalyst supported on titania was prepared using the conventional wet impregnation method by co-impregnating Pd:In in a 1:0.25 wt.% ratio relative to the support (TiO_2_), followed by calcination and reduction.

A solution of PdCl_2_ (Sigma Aldrich, St. Louis, MO, USA, p.a.) and InCl_3_ (Sigma Aldrich, 99.9%) was utilized to achieve the desired bimetallic catalyst. The process involved the addition of a specific mass of TiO_2_ support (Degussa, Zürich, Germany, P25, 48 m^2^/g) to a container containing water, along with a volume of concentrated Pd and In solutions, to attain the desired weight percentages of the metals, namely 1.00% Pd and 0.25% In.

Once the mixture was homogeneous and the solvent was evaporated, the material was dried overnight at 80 °C, and then calcined at 500 °C for 4 h. Finally, it was reduced using a 0.2 M solution of hydrazine hydrate and washed several times with deionized water. The material was left to dry overnight at 80 °C and named PdIn/TiO_2_.

### 3.2. Physicochemical Characterization

X-ray diffraction (XRD), energy-dispersive X-ray spectroscopy (EDX), N_2_ adsorption–desorption isotherms, scanning electron microscopy (SEM), and transmission electron microscopy (TEM) were employed for the physicochemical characterization of catalysts.

XRD powder spectra were generated utilizing the X’Pert PRO X-ray diffractometer (PANalytical, Tokyo, Japan) to ascertain the crystal structure. The measurements were conducted using CuKα radiation (λ = 1.5405 Å) and the X’pert high score plus diffraction software, version 1.0f. The 2θ data were collected in the range of 20° to 100° with a scanning rate of 0.04° s^−1^. The identification of crystalline phases was achieved by comparing the experimental diffraction patterns with those in the Joint Committee on Powder Diffraction Standards (JCPDS).

Morphological characterization of the synthesized catalysts was performed using SEM images recorded with a ZEISS EVO 15 SEM with a 2 nm resolution and Oxford X-MAX 50 mm^2^ EDX.

TEM studies were conducted using a JEOL JEM 2100 electron microscope operating at 100 kV. The samples were diluted in ethanol and placed in a conventional TEM copper grid with a thin holey carbon film.

N_2_ adsorption–desorption isotherms of the carbon supports were measured at −196 °C using Micromeritics ASAP 2020 equipment. The total surface area was calculated from the BET (Brunauer, Emmett, and Teller) equation, and the total pore volume was determined using the single-point method at P/P_0_ = 0.99. Pore size distribution (PSD) curves were obtained from the analysis of the desorption branch of the N_2_ isotherm using the BJH (Barrett, Joyner, and Halenda) method. 

### 3.3. Photochemical Properties

The materials were initially characterized using diffuse reflectance to obtain the band-gap values of the catalysts and narrow down the spectrum of catalysts to be studied. The band-gap values of each material were obtained using the Kubelka–Munk method (K–M or *F*(*R*)), as shown in Equation (12):

(12) $$F\left(R\right)=\frac{(1-R{)}^{2}}{2R},$$

where R is the reflectance, and *F*(*R*) is proportional to the extinction coefficient (α). A modified K–M function can be obtained by multiplying the *F*(*R*) function by hν, using the corresponding coefficient (*n*) associated with an electronic transition (Equation (13)):

(13) $$(F\left(R\right)\times hv{)}^{n}.$$

Graphing Equation (12) as a function of energy in eV yields the value of the material’s band gap. The band gap refers to the energy difference between the valence band (the highest energy level filled with electrons) and the conduction band (the lowest empty energy level) in a material. The size of the band gap determines a material’s ability to absorb light and participate in photochemical reactions. Therefore, materials with smaller band gaps are usually more efficient at utilizing a wider range of light energy, requiring less energy to promote electrons to the conduction band.

### 3.4. Electrochemical Characterization

A temperature of 20 °C was chosen to assess the electrochemical performance of the catalysts in a three-electrode cell controlled via a GAMRY Reference 620–45080 Potentiostat/Galvanostat. The reference electrode used was a reversible hydrogen electrode (RHE), and all potentials mentioned below are presented relative to this electrode. The counter electrode (CE) consisted of a glassy carbon (GC) rod, while the working electrode (WE) was applied as ink onto a GC disk. Assays in a rotating disk electrode (RDE) AUTOLAB RDE-2 were carried out under the same conditions. Current density values were obtained from the geometrical area of the WE.

For the preparation of the inks to be deposited on the GC disk, 2 mg of the catalyst was placed in an Eppendorf tube. Subsequently, 15 μL of NAFION and 500 μL of isopropyl alcohol were introduced into the tube, and the blend was subjected to 30 min of sonication for homogenization. After achieving homogeneity, the dispersed ink (40 μL) was applied onto a polished GC disk (10 mm diameter). The ink was then dried under an inert atmosphere before being utilized in the electrochemical cell. For assays in the RDE, 12 μL of the dispersed ink was applied onto the polished GC disk (3 mm diameter).

The electrochemical behavior of the catalyst powders in a phosphate buffer solution with and without PTM (100 mg·L^−1^), purged with pure N_2_ before each measurement, was examined using cyclic voltammetry (CV) and chronoamperometry techniques.

Electrolytic solutions were prepared using potassium phosphates salts (H_2_KPO_4_ and HK_2_PO_4_) and milli-Q water to form a solution of 0.1 mol·L^−1^ with pH = 7.

Electroactive surface area was estimated from the CV curves of the catalyst at different scan rates in the electrolyte support. CVs were performed for each material, including the bare electrode (i.e., glassy carbon), at different scan rates (5, 10, 20, 50, and 100 mV·s^−1^) in the double-layer region to obtain the electroactive surface area (ECSA). The calculation of *ECSA* (Equation (14)) from the CV data involves the use of the electrochemical double-layer capacitance (EDLC), which can be obtained from the slope of the current density versus scan rate:

(14) $$ECSA=R_{f}\times S$$

Assuming that *S* is the geometric area of 0.785 cm^2^, and *R_f_* is the roughness factor obtained through Equation (15):

(15) Rf=Cdl40 μF·cm−2

Hydrodynamic voltammetry employing Rotating Disk Electrode (RDE) techniques was conducted. The rotation rate of the disk ranged from 750 rpm to 1750 rpm.

### 3.5. Photoelectrochemical Characterization

A Light source, Xe lamp XSS-5XD (Power 150 to 320 W, Radiant Output: 50 W), was used to assess the photoelectrochemical characterization of the materials. A light intensity of 57,500 lux (lumen/m^2^) was used for the experiments.

Photoelectrochemical properties were evaluated using chronoamperometry and CV techniques, both in the presence and absence of irradiation. The tests were carried out in a system as shown in the design shown in Figure S5. The temperature of the working solution was monitored throughout the experiments, and no variations were discerned.

## 4. Conclusions

A small amount of Pd (1.00 wt.%) and In (0.25 wt.%) deposition into TiO_2_ enhanced the light absorption capacity and led to a notable improvement of the hydrogen evolution reaction (HER). This improvement is observed not only in the electrolyte but also in the presence of paracetamol (PTM). In the context of PTM oxidation, both TiO_2_ and PdIn/TiO_2_ exhibit irreversible behavior, primarily hindered by the adsorption of species on the electrode surface. The presence of radiation at TiO_2_-based electrodes completely inhibits the pathway toward soluble species, resulting in a fully irreversible process and improving the catalyst performance toward PTM photoelectroxidation.

Thus, a small amount of Pd and In into TiO_2_ not only increases the (photo)electrocatalytic efficiency toward the PTM oxidation but also intensely raises the HER, which is not inhibited in the presence of the organic molecule, highlighting its capability for both environmental remediation and sustainable hydrogen production.

Consequently, PdIn-doped TiO_2_ emerges as a promising catalyst, showcasing heightened physicochemical properties and superior catalytic performance. This highlights its potential for applications in both environmental remediation and sustainable hydrogen production.

## Figures and Tables

**Table 1:** Double-layer capacitance and electroactive surface area for the photoelectrocatalysts prepared.

Photocatalyst	EDLC_A_ (mF/cm^2^)	EDLC_C_ (mF/cm^2^)	Electroactive Surface Area (cm^2^)
GC	0.00003	−0.00003	0.6
TiO_2_	0.00011	−0.0001	2.5
PdIn/TiO_2_	0.0004	−0.0004	8.24

## Data Availability

The data presented in this study are available in article and Supplementary Materials.

## Supplementary Materials

The following supporting information can be downloaded at https://www.mdpi.com/article/10.3390/molecules29051073/s1, Figure S1. Particle size distribution of TiO_2_ and Pd obtained from counts of different regions and TEM micrographs. Figure S2. (a) Nitrogen adsorption/desorption isotherms of TiO_2_ and PdIn/TiO_2_. (b) BET surface area plot of TiO_2_ and PdIn/TiO_2_. Figure S3. (a) Cyclic voltammograms recorded at different sweep rates 5 (black line), 10 (red line), 20 (green line), 50 (blue line) and 100 (pink line) mV·s^−1^ at GC electrode in 0.1 M phosphate buffer solution. (b) Current density (mA·cm^−2^) as a function of scan rate (V·s^−1^). Data acquired from Figure S3a at 0.3 V. Figure S4. Linear fit of current peak vs square root of scan rate (a) and diffusion current vs the inverse of square root rotation rate (b). Data acquired from Figure 8a,b, respectively. Figure S5. Illustration of the system (not to scale) used for photoelectrochemical assays. (A) Photoelectrochemical cell with four holes for RE, WE, AE, and recirculation of inert gas. (B) Arrangement of cell and lamp spaced 2 cm apart. (C) Arrangement for the RDE system.
