# Supplementary material for: Pd:In-Doped TiO2 as a Bifunctional Catalyst for the Photoelectrochemical Oxidation of Paracetamol and Simultaneous Green Hydrogen Production
Source: Molecules. 2024 Feb 29;29(5):1073. doi: 10.3390/molecules29051073 (PMC10934893; doi:10.3390/molecules29051073)
Supplement: Supplementary file 1 [file molecules-29-01073-s001.zip › molecules-2861129-supplementary.pdf]

SUPPLEMENTARY INFORMATION

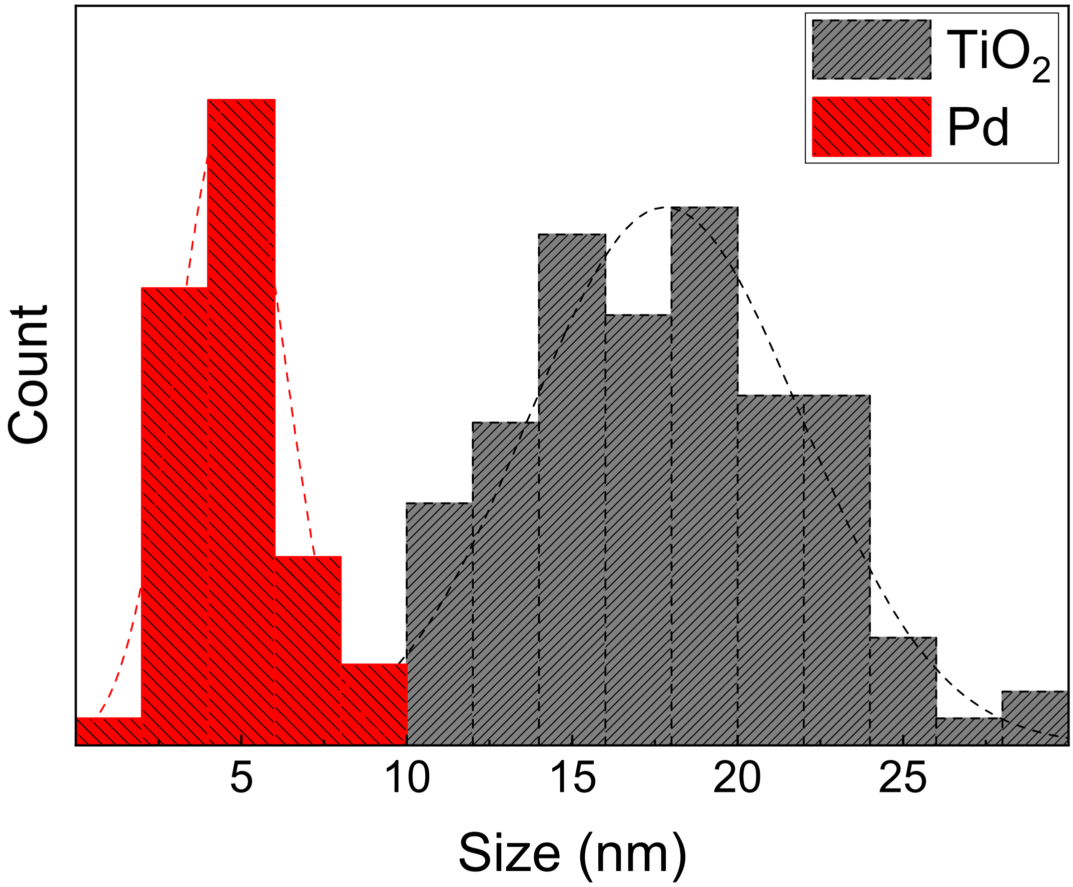

Figure S1. Particle size distribution of TiO_2 and Pd obtained from counts of different regions and TEM micrographs.

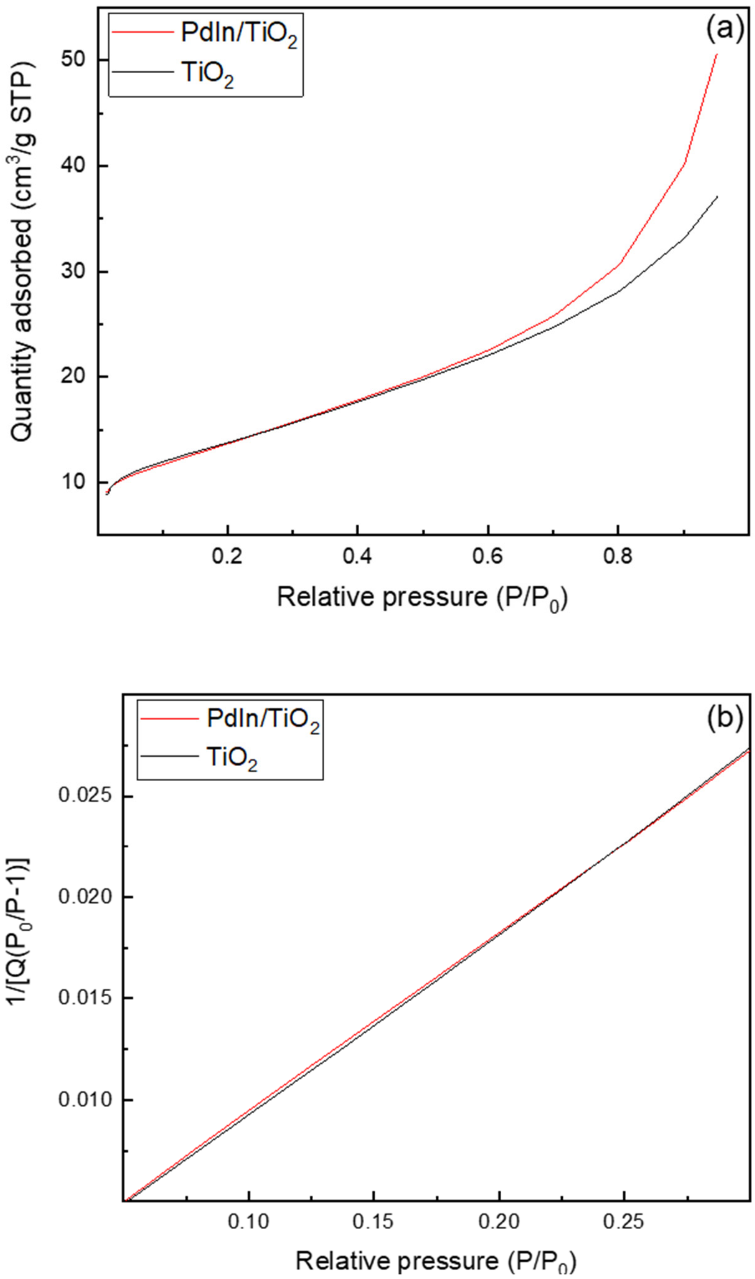

Figure S2. (a) Nitrogen adsorption/desorption isotherms of TiO_2 and $PdIn/TiO_2$. (b) BET surface area plot of TiO_2 and $PdIn/TiO_2$.

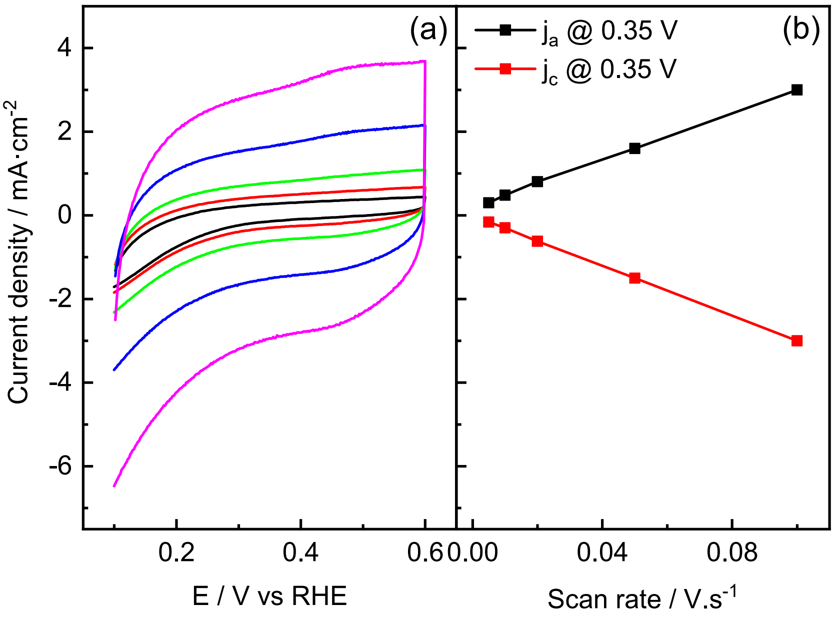

Figure S3. (a) Cyclic voltammograms recorded at different sweep rates 5 (black line), 10 (red line), 20 (green line), 50 (blue line) and 100 (pink line) $mV.s^{-1}$ at GC electrode in 0.1 M phosphate buffer solution. (b) Current density ($mA.cm^{-2}$) as a function of scan rate ($V.s^{-1}$). Data acquired from Fig. S3a at 0.3 V.

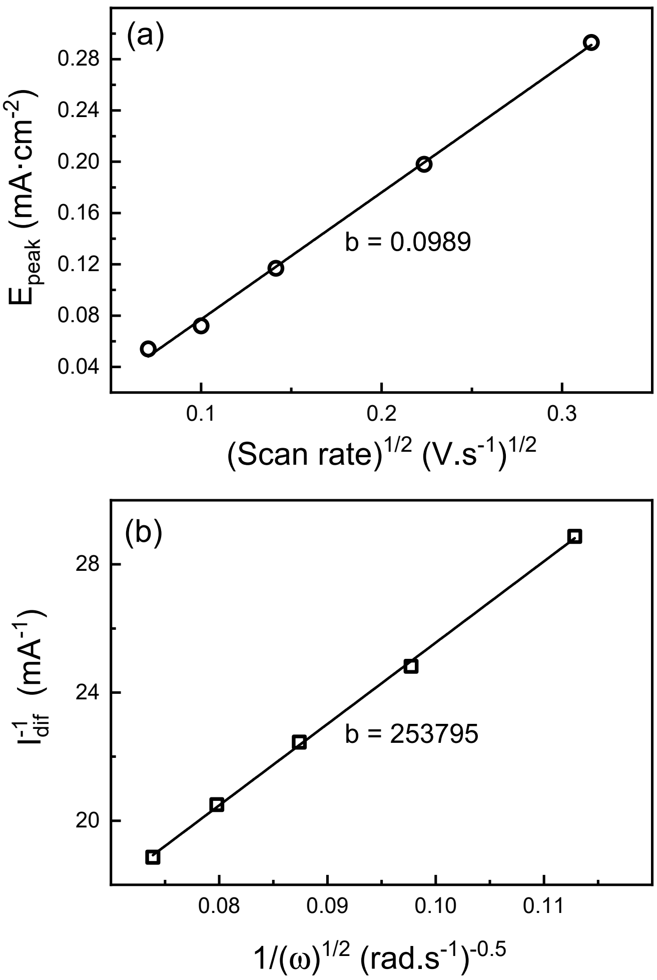

Figure S4. Linear fit of current peak vs square root of scan rate (a) and diffusion current vs the inverse of square root rotation rate (b). Data acquired from Fig. 8.a and 8.b, respectively.

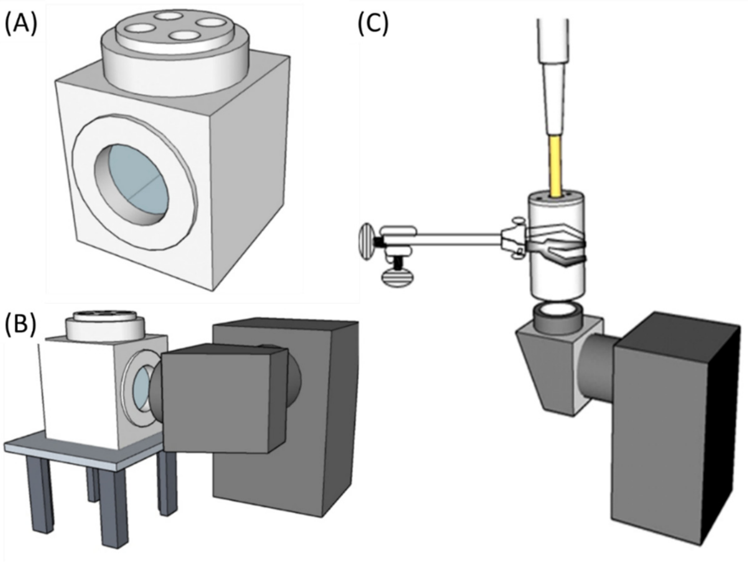

Figure S5. Illustration of the system (not to scale) used for photoelectrochemical assays. (A) Photoelectrochemical cell with four holes for RE, WE, AE, and recirculation of inert gas. (B) Arrangement of cell and lamp spaced 2 cm apart. (C) Arrangement for the RDE system.
